# Authors’ Responses to Peer Reviews of “Utility of the ROX Index in Predicting Intubation for Patients With COVID-19–Related Hypoxemic Respiratory Failure Receiving High-Flow Nasal Therapy: Retrospective Cohort Study”

**DOI:** 10.2196/31892

**Published:** 2021-08-27

**Authors:** Maulin Patel, Junad Chowdhury, Nicole Mills, Robert Marron, Andrew Gangemi, Zachariah Dorey-Stein, Ibraheem Yousef, Matthew Zheng, Lauren Tragesser, Julie Giurintano, Rohit Gupta, Parth Rali, Gilbert D'Alonzo, Huaqing Zhao, Nicole Patlakh, Nathaniel Marchetti, Gerard Criner, Matthew Gordon

**Affiliations:** 1 Department of Thoracic Medicine and Surgery Temple University Hospital Philadelphia, PA United States

**Keywords:** respiratory, medicine, nasal therapy, COVID-19, mechanical ventilation, ventilators, mortality, morbidity, intubation

*This is the authors’ response to peer-review reports for the paper “Utility of the ROX Index in Predicting Intubation for Patients With COVID-19–Related Hypoxemic Respiratory Failure Receiving High-Flow Nasal Therapy: Retrospective Cohort Study”.*

## Round 1 Review

### Reviewer G

#### Specific Comments

##### Major Comments

Thank you, Reviewer G [1], for your comments on our paper [2]. We appreciate your wonderful feedback.

1. The Methods section was modified to clarify the inclusion criteria further. There were two stages to our screening process; hence, the inclusions and exclusions were written to reflect a step-by-step method of achieving the final N. The CONSORT (Consolidated Standards of Reporting Trials) diagram at the end further clarifies the process.
2. The 35 L/min flow was the starting point for the HFNT (high-flow nasal therapy) initiation protocol. Immediately, adjustments were made based on the patient’s tolerance and oxygenation. The “33.5, SD 11.7” value in the Results section is the average first flow rate documented in our Electronic Medical Record (EMR).
3. The first two paragraphs were written as a summary of the overall results as stated by the journal guidelines for the discussion. We revised the first two paragraphs to make the summary more concise.

##### Minor Comments

1. The error was corrected.
2. Those with high clinical suspicion were indeed negative by PCR (polymerase chain reaction). This was added to the Methods section.
3. The confidence interval was 0.994 to 4.591. We adjusted the language of the paper to reflect the above results in a more appropriate way.
4. In other words, our analysis showed that any lack of improvement or negative change in ROX (ratio of oxygen saturation) index was predictive of intubation. The sentence was rewritten to explain this better.
5. We will change the reported values to AUC (area under the receiver operating characteristic curve) in the paper.

### Reviewer R

#### Major Comments

Thank you, Reviewer R [3], for your wonderful comments. We appreciate your feedback.

1. We have changed the objective to be distinguishable from the key question.
2. The paragraphs were separated to highlight the two sections.
3. We refined the Methods section with the goal of improving it. The subsections were redefined to improve this section. The treatment protocols will be moved to the supplementary materials section. We are happy to redact more, if necessary.
4. This was a retrospective observational study. Hence, the authors made no contribution to the actual treatments of patients. We used the data available to us afterward to evaluate the ROX index. It was not until we had analyzed our data that we started using the ROX index in our intensive care unit routinely.
5. Noted. Changes have been made as per the suggestions.
6. Our Discussion section includes a section specifically on strengths and limitations. We are happy to separate it out as a different section, if needed.
7. The ROX index is a noninvasive score that can easily be applied at any hospital without the addition of any new parameters. It includes pulse oximetry, fraction of inspired oxygen, and respiratory rate. All hospitals will always have these parameters available to them. Thus, ROX gives physicians a noninvasive tool during a pandemic when minimizing exposure is key to preventing transmission.
8. Our figures were submitted separately from the main submission per the submission guidelines. We will include all the images with the main document in the revised version.
9. We are happy to provide our data analysis to the reviewer separately, if needed. We feel discussing all the details of how we generated our results step by step will dilute the importance of the results highlighted in the Results section. Moreover, we feel this might not be ideal for a reader who has only a basic statistical background.
10. All the results mentioned in the paper have been presented in textual and graphical forms (graphs and tables), wherever applicable. The majority of the discussion involves a review of previous data and an explanation of our results, which will be difficult to write in a tabular form. We are happy to rearrange portions in a tabular format if the reviewer would be kind enough to point out a specific section.
11. As mentioned above, we are happy to provide all the derivative equations to the reviewer if that helps. However, we felt that some of these derivatives are complex and take away from the results of the paper. Moreover, the majority of studies written usually do not provide the actual calculations of their results. Most studies have the data analysis available upon request, which we are happy to provide.
12. Thank you.
13. Thank you for the wonderful feedback.

#### Minor Comments

1. The majority of typos and grammatical issues have been corrected.
2. The article was restructured according to the points made by the reviewer.
3. We will review the guidelines again and try to conform to those guidelines.
4. The images will be made available at the end of the paper per the submission guidelines.
5. We are happy to provide data sets upon request. We do not want our data sets to be publicly available, but we are happy to share them on a case-by-case basis. We do mention this in our paper.
6. We corrected this section to accommodate your request.
